# The spatial range of peripheral collinear facilitation

**DOI:** 10.1038/srep15530

**Published:** 2015-10-27

**Authors:** Marcello Maniglia, Andrea Pavan, Felipe Aedo-Jury, Yves Trotter

**Affiliations:** 1 Université de Toulouse-UPS, Centre de Recherche Cerveau et Cognition, Toulouse, France; 2 Centre National de la Recherche Scientifique, Toulouse Cedex, France; 3 University of Lincoln, Brayford Pool, Lincoln, Lincolnshire, LN6 7TS, United Kingdom

## Abstract

Contrast detection thresholds for a central Gabor patch (target) can be modulated by the presence of co-oriented and collinear high contrast Gabors flankers. In foveal vision collinear facilitation can be observed for target-to-flankers relative distances beyond two times the wavelength (λ) of the Gabor’s carrier, while for shorter relative distances (<2λ) there is suppression. These modulatory influences seem to disappear after 12λ. In this study, we measured contrast detection thresholds for different spatial frequencies (1, 4 and 6 cpd) and target-to-flankers relative distances ranging from 6 to 16λ, but with collinear configurations presented in near periphery at 4° of eccentricity. Results showed that in near periphery collinear facilitation extends beyond 12λ for the higher spatial frequencies tested (4 and 6 cpd), while it decays already at 10λ for the lowest spatial frequency used (i.e., 1 cpd). In addition, we found that increasing the spatial frequency the peak of collinear facilitation shifts towards larger target-to-flankers relative distances (expressed as multiples of the stimulus wavelength), an effect never reported neither for near peripheral nor for central vision. The results suggest that the peak and the spatial extent of collinear facilitation in near periphery depend on the spatial frequency of the stimuli used.

Surrounding elements influence the detection and discrimination of a localized target^1^,^2^. There is psychophysical evidence that the contrast detection threshold of a Gabor stimulus can be either increased or reduced by the presence of co-oriented and collinear high contrast Gabor flankers having the same spatial frequency and spatial phase of the target^3^,^4^,^5^. Lateral interactions have been investigated in terms of orientation selectivity^4^,^5^, spatial frequency selectivity^6^ and target-to-flankers relative distances for collinear facilitation^4^,^5^; that is, the target-to-flankers relative distance for which the contrast detection threshold of the central target is reduced^3^. In general, collinear facilitation is observed when flankers are located beyond two times the Gabor carrier’s wavelength (λ) while for shorter distances (<2λ) there is suppression, meaning that the contrast detection thresholds for the central target are higher. It should be noted that in the collinear facilitation literature, distances defined in λ are multiples of the carrier’s wavelength and do not indicate angular distance per se, which is derived by the number of λ and the spatial frequency of the stimulus.

It has been proposed that the anatomical substrates of collinear facilitation are the long-range connections between neurons with similar orientation selectivity in the primary visual cortex (V1)^7^,^8^,^9^,^10^,^11^,^12^,^13^. This hypothesis is supported by a psychophysical estimation of the propagation speed of collinear facilitation^12^, that is consistent with measurement from intracellular recording of horizontal connection in V1^13^. The estimated propagation speed of collinear facilitation is ~3 deg/s, that is much slower than feedback and feed forward connections in early visual areas^14^ and it is consistent with temporal dynamics of lateral conduction of sub-threshold activity elicited by the surround^9^,^13^,^15^. Additionally, physiological studies on integration of responses to a visual target indicate a time window of 200 ms^10^, in agreement with the integration window for contrast detection^16^. Consistently, Polat and colleagues^17^ showed that contrast sensitivity increases from presentation duration of 30 ms to 120 ms, reaching saturation for longer presentation times. Therefore, an input that arrives after a time window of 100–200 ms will no longer be contributing to the target’s response.

Furthermore, there is physiological evidence that horizontal connections are likely to be involved in a variety of contextual modulatory effects: a) in macaque monkeys, inhibition (cooling) of area V2 has no effect on the response to a static texture surround in V1 units^18^; b) horizontal connections seem to be denser than the feedback projections^19^; c) both long range striate^8^,^20^,^21^,^22^,^23^ and extrastriate^7^,^24^ connections are present between units tuned for similar orientation; this is in contrast with Stettler *et al.*^19^ who reported that V2–V1 feedback do not occur between units with similar orientation selectivity.

However, collinear facilitation might be also modulated by the activity of higher-level, extrastriate visual areas (e.g., V2, V4). It is well known that the activity of V1 can be modulated by feedback connections controlling the response gain of its target neurons^25^. There is physiological evidence that inhibition of the extrastriate area V2 produces a decrement of V1 responses^14^,^26^. Moreover, neurons in higher-level visual areas present a larger receptive field than V1 neurons, thus pooling information over a larger visual area. Indeed, cell recording in macaque monkeys showed that V2 neurons pool information from an area 5–6 times larger than the integration area of V1 cells^27^.

On the other hand, other studies do not support the idea that horizontal connections are solely responsible for contextual modulatory effects. For example, in area V1 at 2.5° of eccentricity, an axonal length of 3 mm corresponds to 0.5° in the visual field^28^, an angular distance much smaller than the extent of modulatory interaction in V1^29^. Consistently, feedback connections, not strictly limited by retinotopy, can gather information from regions that are more far away in the visual field, so they can be a plausible candidate for long-range modulatory effects.

In general, it is plausible that both horizontal and feedback connections contribute to collinear facilitation, with horizontal connections mediating near interactions, such as those located within the summation field, while feedback connections modulate responses to contextual elements located at far distance^27^,^30^,^31^,^32^.

Overall, these context-related effects seem to be mediated by interactions between cells with a classical receptive field (CRF), organized in hypercolumns and selective for basic features such as orientation, retinal location, spatial phase and spatial frequency.

Similarly to the physiological structure of striate cells, composed by a central excitatory subunit with an antagonistic surround, a proposed corresponding basic unit in visual perception is the Perceptual Field (PF) made up by spatial filters with 2–3 antagonistic elements^33^,^34^ with a main component and one or more smaller elements.

Consistent with the role of the CRF in the cytoarchitecture of the visual cortex, the PF represents the first ‘brick’ in the construction of the visual percept. Indeed, previous studies on lateral interactions with Gabor stimuli indicated that collinear facilitation and inhibition mechanisms result from between and within PF stimulation, for facilitatory and suppressory effects, respectively^3^,^35^. Therefore, the distance between target and flankers that produces suppression can be used to estimate the size of the PF psychophysically; a question still debated in the case of stimulus presentation in near peripheral vision^36^. The seminal study of Polat and Sagi^3^ reported that the foveal range of lateral interactions extends up to 12λ for mid-high spatial frequencies (6–13 cpd), while for flankers placed beyond this distance, the modulation of contrast is marginal or absent^4^,^5^,^11^. Additionally, there is electrophysiological evidence in humans that visually evoked potentials (VEPs) amplitude is maximal for target-to-flankers distances of 1 degree of visual angle for both collinear and orthogonal configurations (i.e., a baseline configuration with flankers orthogonally oriented with respect to the vertical central target)^11^. Since Polat and Norcia^11^ used Gabor stimuli with a spatial frequency of 3 cycles per degree (cpd), in their experiment a target-to-flankers distance of 1 deg corresponded to a relative distance of 3λ. This is consistent with psychophysical evidence that the peak of collinear facilitation is located around 3λ^3^. Polat and Norcia^11^ also found that response amplitudes of VEPs were significantly lower for the orthogonal configuration, decreasing as a function of distance. Finally, at 4 deg of target-to-flankers angular distance (corresponding to 12λ), no differences in VEPs responses between collinear and orthogonal configuration were observed. As mentioned earlier, this is the same range of collinear facilitation estimated psychophysically in previous studies^3^.

All the studies on collinear facilitation discussed so far focused on foveal presentation. However physiological evidence from cats and monkeys showed that the long-range connections in the foveal projection of V1 area are a possible neural substrate of this phenomenon that may extend to the peripheral projection area^7^,^21^. Moreover, single cell recordings in monkeys and macaques showed contextual modulatory effects for stimuli located up to 10° of eccentricity^1^,^10^,^13^ involving possibly both horizontal connections and/or feedback connections from higher visual areas^37^. Therefore, physiological evidence seem to indicate that collinear facilitation should be present also for near peripheral presentation of the stimuli. Indeed, a series of recent studies^36^,^38^,^39^ showed that collinear facilitation can be observed in the near-periphery of the visual field (4° of eccentricity), with some differences with respect to the foveal vision: a) it emerges at a target-to-flankers relative distance of 6–8λ^36^,^38^, approximately 2–3 times the minimum separation for eliciting collinear facilitation in fovea (3λ); b) it shows a preference for lower spatial frequencies^39^, consistently with the contrast sensitivity function measured in peripheral vision^40^; c) it is present from a target-to-flankers relative distance of 6λ for stimuli with a spatial frequency of 1 cpd (corresponding to an angular distance of 8 deg), whereas for higher spatial frequencies collinear facilitation emerged at 8λ (corresponding to an angular distance of 4°, 2° and 1.33° for 2, 4 and 6 cpd, respectively^39^; d) the difference in contrast sensitivity between collinear and orthogonal condition at the critical relative distance of 8λ decreases as spatial frequency increases^39^, the opposite of what found in fovea where the magnitude of collinear facilitation (measured at the critical relative distance of 3λ) increases as spatial frequency increases^6^ (differences are summarized in Table 1). However, apart for these few recent studies, collinear facilitation with stimuli presentation in the near-periphery of the visual field has not been properly investigated. In particular, there are no studies on the extent of the range of collinear interactions (expressed as multiples of the stimulus wavelength) in near periphery. Maniglia and colleagues^39^ tested up to 8λ, while earlier investigations did not test beyond 7λ^36^,^41^.

The aim of the present study was to assess the spatial range of collinear facilitation with stimuli presented at 4° of eccentricity for different spatial frequencies.

The first hypothesis, arising from recent studies on near peripheral collinear facilitation^36^,^38^, proposed that the overall range of collinear facilitation would be shifted towards higher target-to-flankers relative distances, with the expected decay of collinear facilitation located beyond the foveal limit of 12λ. This hypothesis is consistent with the idea that PFs increase in size with eccentricity^36^ and fits with previous studies^41^,^42^ in which testing target-to-flankers relative distances that are facilitatory in fovea (3–4λ) led to suppression. This suppression is likely due to the 3-elements collinear configuration still falling within the same PF.

The second hypothesis proposed that the facilitatory range of collinear facilitation (i.e., the absolute length of spatial interaction) would be larger. This because each unit responding to flanking stimuli would have a larger PF and consequently would be activated by stimuli located at farther spatial locations, consistently with the cortical magnification factor^43^.

The third hypothesis, based on Maniglia *et al.*^39^, proposed that different spatial frequencies might show different facilitation curves. In particular, low spatial frequencies might have either an overall larger facilitatory range, starting from 6λ and decaying at the same target-to-flankers distance as for higher spatial frequencies; or it could be shifted leftwards with respect to the higher spatial frequencies, decaying at shorter relative distances.

In order to test these three hypotheses, we measured near peripheral collinear facilitation with one low spatial frequency (1cpd), two intermediate spatial frequencies (4 and 6 cpd) and a large range of target-to-flankers relative distances (i.e., from 4λ to 16λ). In order to obtain a reliable baseline measurement, we measured contrast thresholds also in the orthogonal condition; with flankers orthogonally oriented to the target, a configuration that does not elicit collinear interactions^3^,^11^ and is commonly used as baseline condition for measuring collinear facilitation^38^,^39^,^41^.

Concerning the first hypothesis, the facilitation range in near periphery appeared to be overall shorter than the facilitation range reported in fovea. The difference between collinear and orthogonal thresholds was not statistically significant at 12λ for 4 and 6 cpd and at 10λ for 1cpd. However, if we consider the facilitation range in a broader term, as the target-to-flankers relative distance at which the facilitation decays completely (i.e., the distance at which the average contrast thresholds for the collinear condition are equal or higher than those of the baseline condition)^3^, we observe that the facilitation range for 4 cpd decays completely at 16λ, while for 6 cpd it seems to extend beyond that distance.

For the second hypothesis, when compared to the foveal range of lateral interactions (i.e., from 2λ to 12λ), collinear facilitation in the near periphery does not overcome this range, instead it seems to be shorter, especially for the lowest spatial frequency tested (1cpd).

Consistently, concerning the third hypothesis, it seems that collinear facilitation in the near periphery depends at least in part on the spatial frequency, with an overall extent of collinear interactions larger for the higher spatial frequencies tested (8λ–14λ and 8λ–16λ for 4cpd and 6cpd, respectively) and shorter for the lowest spatial frequency (6λ–12λ for 1 cpd).

In addition, the peak of collinear facilitation seems to be shifted towards larger target-to-flankers relative distances for higher spatial frequencies. This phenomenon has never been reported neither for near peripheral nor for foveal presentations. Previous studies on foveal collinear facilitation assumed that the target-to-flankers relative distance at which the peak of facilitation is reached is independent from spatial frequency^3^,^6^. The rationale was that 3λ is the shortest relative distance at which flankers fall outside the PF responding to the target, eliciting in turn modulation between (and not within) PFs.

## Experiment 1

The aim of Experiment 1 was to measure the spatial range of collinear facilitation in the near periphery (4° of eccentricity) with low spatial frequency Gabor patches (i.e., 1 cpd). In particular, we assessed the decay of collinear facilitation using a range of target-to-flankers relative distances (i.e., from 6λ to 12λ).

## Methods

### Apparatus

Stimuli were displayed on a 17” Dell M770 CRT monitor with a refresh rate of 60 Hz. We generated the stimuli with Matlab Psychtoolbox^44^,^45^. The screen resolution was 1024 × 768 pixels. Each pixel subtended 1.9 arcmin. The minimum and maximum luminance of the screen were 0.98 cd/m^2^ and 98.2 cd/m^2^, respectively, and the mean luminance was 47.6 cd/m^2^. Luminance was measured with a Minolta CS110 (Konica Minolta, Canada). A digital-to-analogue converter (Bits#, Cambridge Research Systems, Cambridge UK) was used to increase the dynamic contrast range (12-bit luminance resolution). A 12-bit gamma-corrected lookup table (LUT) was applied so that luminance was a linear function of the digital representation of the image.

### Participants

Two authors (MM and FAJ) and eight naïve observers took part in the experiment. All participants had normal or corrected to normal visual acuity. They sat in a dark room at a distance of 57 cm from the screen. The participant’s head was stabilized using a chinrest. Viewing was binocular. They were instructed to fixate at the center of the screen where a fixation point was always present. All participants took part voluntarily. In addition, all participants gave written informed consent prior to their inclusion in the experiment. This study was conducted in accordance with the Declaration of Helsinki (1964). The experimental protocol was approved by the relevant ethical committee at Centre National de la Recherche Scientifique with our institutional review board (CPP, Comité de Protection des Personnes, protocole 13018–14/04/2014).

### Gabor stimuli

**Figure 1:**
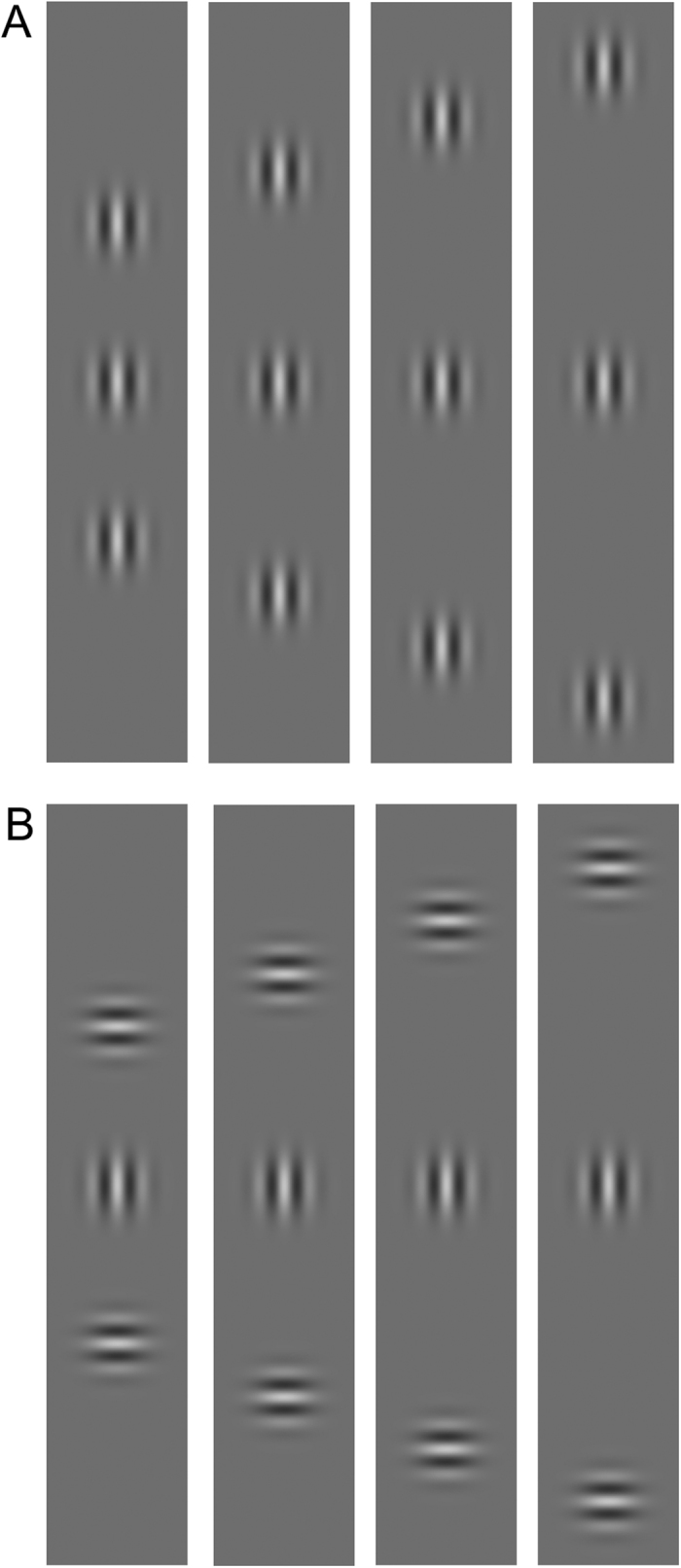
Stimuli used in Experiment 1. (**A**) Collinear configurations of 1 cpd with target-to-flankers relative distances of 6λ, 8λ, 10λ and 12λ. (**B**) Orthogonal configurations of 1 cpd with a target-to-flankers relative distance of 6λ, 8λ, 10λ and 12λ. The contrast of the central Gabor patch (i.e., the target) is increased for demonstrative purposes.

Stimuli were Gabor patches consisting of a cosinusoidal carrier enveloped by a stationary Gaussian and arranged vertically (Fig. 1). The luminance distribution of the Gabor signal was defined as:


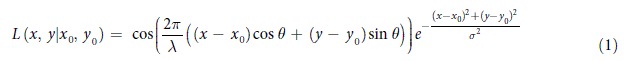


where *x* is the horizontal axis, y the vertical axis, *θ* is the orientation of the Gabor patch (in radiants), λ is the wavelength of the cosinusoidal carrier, and σ is the standard deviation of the Gaussian envelop. In all experiments σ = λ^3^. Gabor patches had a spatial frequency of 1 cpd. The location of the target relative to the fixation point (0.18 deg) was 4° either in the left or in the right visual hemi-field. A vertical Gabor target was presented flanked above and below by two high-contrast Gabor patches (0.6 Michelson contrast) (Fig. 1). In the collinear configuration target and flankers were vertically oriented (Fig. 1A), whereas in the orthogonal configuration flankers were orthogonally oriented with respect to the target (Fig. 1B). Flankers were located at various distances from the target.

### Procedure

**Figure 2:**
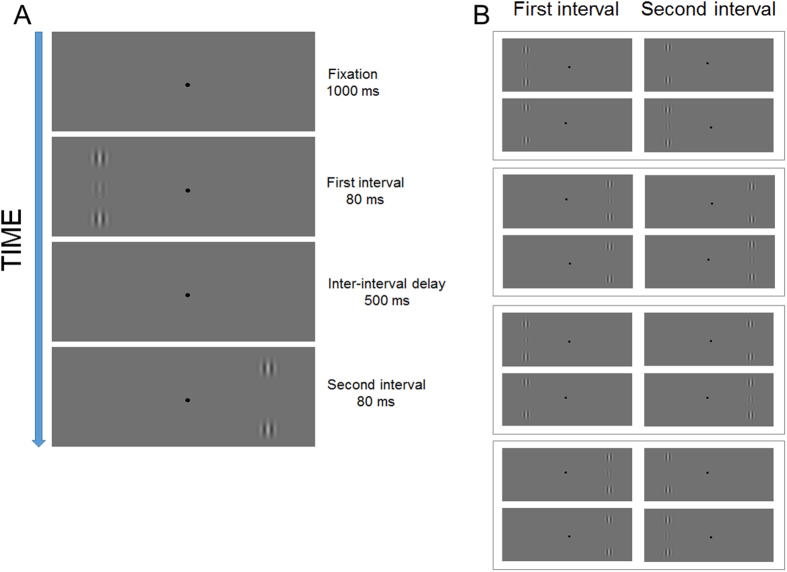
Schematic representation of the procedure used in Experiment 1. (**A**) In the example, the target is shown in the first temporal interval and left visual hemi-filed, whereas only the flankers are displayed in the second temporal interval and in the right visual hemi-field. (**B**) All the possible combinations of temporal intervals and presentation visual hemi-fields.

Lateral interactions were assessed by comparing the contrast detection thresholds estimated in the collinear and orthogonal configurations as a function of the target-to-flankers relative distance (4λ, 5λ, 6λ, 8λ, 10λ, 12λ, and 14λ). In Experiment 1 the spatial frequency tested was 1 cpd. Contrast detection thresholds were measured at 4° of eccentricity. We used a two-interval forced choice task (2IFC) in which participants were required to choose which of the two temporal intervals contained the target. The target was present only in one interval, while the flankers were presented in both intervals. Each interval was presented for 80 ms with an inter-interval delay of 500 ms^6^,^39^ (Fig. 2A). The target could be presented either in the left or right visual hemi-field. The temporal interval and the visual hemi-field were randomized on a trial basis (see Fig. 2B).

The contrast of the target was varied according to a simple 1up-3down staircase^46^. The starting contrast of the target was set at 0.1 Michelson contrast, increasing of 0.1 log units for each wrong response and decreasing of the same value after three consecutive correct responses. The staircase terminated after either 120 trials or 14 reversals. Contrast thresholds, corresponding to 79% of correct responses, were calculated averaging the contrast values corresponding to the last 6 reversals, regardless the temporal interval in which the target was presented and the presentation visual hemi-field. An acoustic feedback (50 ms tone of 500 Hz) was provided with wrong answers. Observers performed 8 blocks in which the target-to-flankers relative distance and the flankers’ orientation were varied. Observers performed the experiment in one day.

## Results

**Figure 3:**
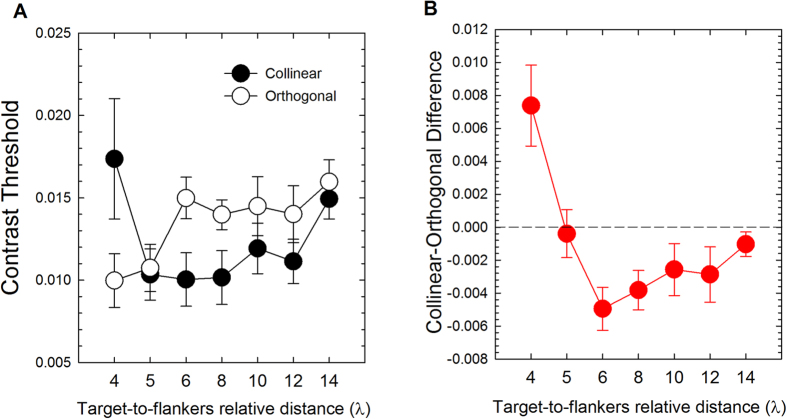
Results of Experiment 1. (**A**) Mean contrast thresholds (Michelson contrast) for collinear and orthogonal configurations as a function of target-to-flankers relative distance. (**B**) Mean differences between collinear and orthogonal contrast thresholds (i.e., collinear-orthogonal difference) as a function of the target-to-flankers relative distance. The horizontal black dashed line represents a difference equal to zero (i.e., no modulation). Data points below zero represent collinear facilitation. Error bars ± s.e.m.

Figure 3 shows the results of Experiment 1. Contrast thresholds (Michelson contrast) and differences between collinear and orthogonal contrast thresholds are shown as a function of the target-to-flankers relative distance.

### Spatial extent of collinear facilitation

To estimate the spatial range of collinear facilitation for 1 cpd stimuli, we conducted a repeated-measures ANOVA including as factors the configuration (collinear vs. orthogonal) and the target-to-flankers relative distance (4λ, 5λ, 6λ, 8λ, 10λ, 12λ, 14λ). The ANOVA did not report a significant effect of the target-to-flankers relative distance (*F*_2.34, 21.09_ = 1.47, *p* = 0.251, *partial-η*^2^ = 0.14, degrees of freedom and *p* value were corrected with the Greenhouse-Geisser correction because of sphericity violation), but reported a marginally significant effect of the configuration (*F*_1,9_ = 4.62, *p* = 0.06, *partial-η*^2^ = 0.34). The interaction between configuration and target-to-flankers relative distance was significant (*F*_6,54_ = 6.65, *p* = 0.0001, *partial-η*^2^ = 0.43). Post-hoc t-tests, using a false discovery rate (FDR) of 0.05^47^, showed that at 4λ the contrast thresholds in the collinear condition was higher than the orthogonal contrast threshold (Table 2, line 1); this is consistent with the idea that for short relative distances, collinear interactions in the near periphery are mostly inhibitory^38^,^41^. On the other hand, collinear thresholds were lower for target-to-flankers relative distance of 6λ and 8λ (see Table 2 and Fig. 3A). These results are consistent with previous studies^36^,^38^,^39^.

Figure 3B shows the mean differences between the contrast thresholds estimated in the collinear configuration and those estimated in the orthogonal configuration for each target-to-flankers relative distance. For the sake of simplicity we will refer to the difference between collinear and orthogonal threshold as “collinear-orthogonal difference”. A repeated-measures ANOVA on the mean collinear-orthogonal differences and including as factor the target-to-flankers relative distance reported a significant effect of the separation (*F*_6,54_ = 6.65, *p* = 0.0001, *partial-η*^2^ = 0.43). Simple within-subjects contrasts showed that the collinear-orthogonal difference at 4λ was significantly different than the collinear-orthogonal differences calculated for all the other target-to-flankers relative distances (Table 3). In addition, reverse Helmert within-subjects contrasts showed a significant difference between the collinear-orthogonal difference at 6λ and 5λ (*F*_*1*,9_ = 13.82, *p* = 0.005, *partial-η*^2^ = 0.61) and a significant difference between the collinear-orthogonal difference at 8λ and 6λ (*F*_*1*,9_ = 7.71, *p* = 0.022, *partial-η*^2^ = 0.46).

Finally, we performed seven two-sided one-sample t-tests using a FDR of 0.05. The t-tests were performed between collinear-orthogonal differences, for each target-to-flankers relative distance, and zero (i.e., no modulation). The t-tests showed that collinear-orthogonal differences were significantly lower than zero for 6λ and 8λ and significantly higher than zero for 4λ (Table 4).

## Experiment 2

The aim of Experiment 2 was to measure collinear facilitation in the near periphery (4° of eccentricity) with mid-high spatial frequencies (i.e., 4 and 6 cpd). In particular, this experiment was conducted to assess whether higher spatial frequencies show a different range of collinear facilitation. In Experiment 1 and in our previous study^39^ we found that for 1 cpd, collinear facilitation emerged at shorter target-to-flankers relative distance than for higher spatial frequencies (i.e., 6λ for 1cpd, ~8λ for 2 and 3 cpd^39^) and vanishes around 10λ (against the 12λ of the fovea^3^). Such reduced range of collinear facilitation might be due to the temporal integration window for the central target^10^, so that inputs coming from the flankers fail to reach the target within the temporal constraint of contrast integration^6^,^12^. Since in the lateral interactions paradigm^3^ lower spatial frequencies would lead to greater angular distances between target and flankers, it is possible that the reduced range of collinear facilitation depends on the failure of temporal signal integration between flankers and target. In order to test whether higher spatial frequencies would lead to a larger collinear facilitation range, we tested collinear facilitation at 4 and 6 cpd for a number of target-to-flankers relative distances.

## Methods

One author (MM) and a new sample of nine naïve observers took part in Experiment 2. Stimuli and apparatus were the same as in Experiment 1. The procedure was the same as used in Experiment 1 except that we used spatial frequencies of 4 and 6 cpd and target-to-flankers relative distances ranging from 6λ to 16λ (i.e., 6λ, 8λ, 10λ, 12λ, 14λ, and 16λ).

## Results

### Extent of collinear facilitation for higher spatial frequency

**Figure 4:**
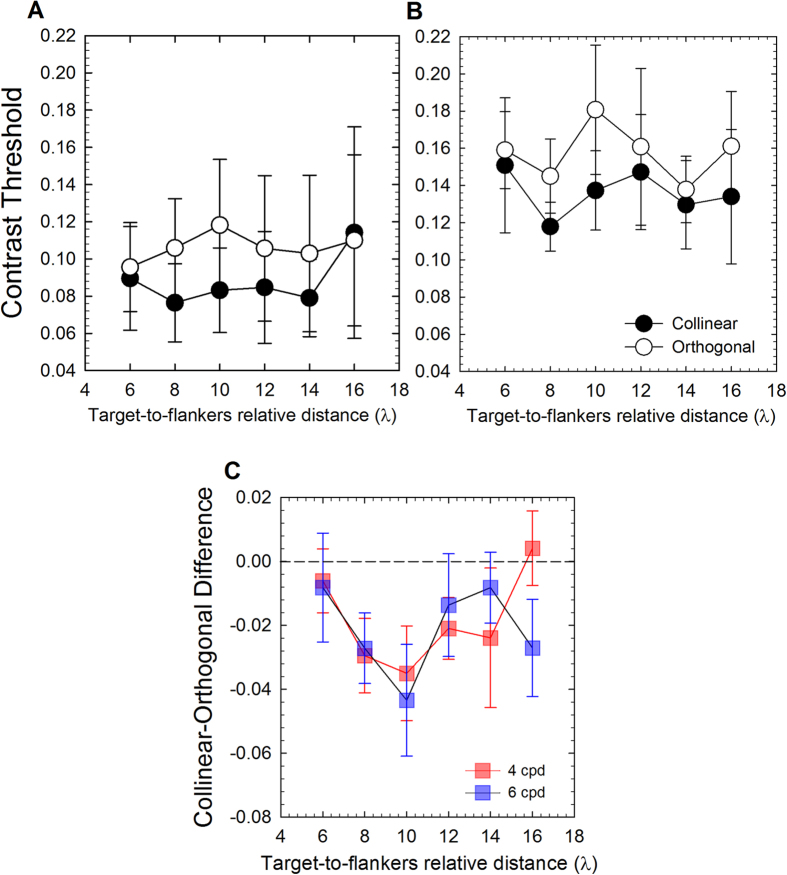
(**A**,**B**) Mean contrast detection thresholds (Michelson contrast) for collinear and orthogonal configurations as a function of the target-to-flankers relative distance for 4 cpd (panel A) and 6 cpd (panel B). (**C**) Mean collinear-orthogonal difference for each target-to-flankers relative distance and for 4 and 6 cpd. The horizontal black dashed line represents a difference equal to zero (i.e., no modulation). Data points below zero represent collinear facilitation. Error bars ± s.e.m.

Figure 4 shows contrast thresholds (panel A) and mean collinear-orthogonal differences (panel B) as a function of the target-to-flankers relative distance. A repeated-measures ANOVA on the contrast thresholds and including as factors the spatial frequency (4 cpd vs. 6 cpd), the configuration (collinear vs. orthogonal) and the target-to-flankers relative distance (6λ, 8λ, 10λ, 12λ, 14λ, 16λ), reported a significant effect of the spatial frequency (*F*_1,9_ = 26.66, *p* = 0.001, *partial-η*^2^ = 0.75) and a significant effect of the configuration (*F*_1,9_ = 9.62, *p* = 0.013, *partial-η*^2^ = 0.52). In addition to better investigate the pattern of lateral interaction for mid-high spatial frequencies we conducted two repeated-measures ANOVA separately for 4 and 6 cpd.

For 4 cpd the ANOVA reported a significant effect of the configuration (*F*_1,9_ = 8.43, *p* = 0.017, *partial-η*^2^ = 0.48), but not a significant effect of the target-to-flankers relative distance (*F*_1.17, 10.6_ = 0.47, *p* = 0.54, *partial-η*^2^ = 0.05) or a significant interaction configuration x target-to-flankers relative distance (*F*_1.6, 14.5_ = 1.35, *p* = 0.28, *partial-η*^2^ = 0.13).

For 6 cpd, the ANOVA also reported only a significant effect of the configuration (*F*_1,9_ = 6.11, *p* = 0.035, *partial-η*^2^ = 0.40), but not a significant effect of the target-to-flankers relative distance (*F*_2.35, 21.2_ = 0.793, *p* = 0.48, *partial-η*^2^ = 0.08) or a significant interaction configuration x target-to-flankers relative distance (*F*_2.21, 19.94_ = 0.66, *p* = 0.54, *partial-η*^2^ = 0.07).

Despite the interaction was not significant, we also conducted a series of paired t-tests between the contrast thresholds estimated in the collinear and orthogonal configurations, separately for each target-to-flankers relative distance^38^,^39^ and spatial frequency. Significant paired t-tests are summarized in Table 5. Overall, the results showed that for both spatial frequencies (i.e., 4 and 6 cpd) the contrast thresholds in the collinear condition were lower for target-to-flankers relative distance of 8λ and 10λ. In addition, the difference between contrast thresholds estimated in the collinear and orthogonal configurations at 4 cpd for 12λ was close to significance (*t*_(9)_ = −2.20, *p* = 0.055, *d* = 0.69).

Additionally, we conducted a repeated-measures ANOVA, separately for 4 and 6 cpd, on the collinear-orthogonal differences including as factor the target-to-flankers relative distance. The ANOVA did not report a significant effect of the target-to-flankers relative distance for both spatial frequencies (4 cpd: *F*_1.6, 14.5_ = 1.35, *p* = 0.28, *partial-η*^2^ = 0.13; 6cpd: *F*_2.16,19.5_ = 0.66, *p* = 0.54, *partial-η*^2^ = 0.068). Finally, we also compared the collinear-orthogonal differences with respect to zero. The results showed that for both spatial frequencies (i.e., 4 and 6 cpd) collinear-orthogonal differences were significantly lower than zero at 8λ and 10λ (Table 6). In addition, for 4 cpd the difference between threshold elevation and zero at 12λ was close to significance (*t*_(9)_ = −2.20, *p* = 0.055, *d* = 0.69).

### Peak of collinear facilitation

There is psychophysical evidence that foveal collinear facilitation peaks at target-to-flankers relative distance of 3λ^3^,^4^,^5^,^11^, and this regardless the spatial frequency used^6^. In an additional analysis we fitted a lognormal function on the mean collinear-orthogonal differences in order to estimate the peak of collinear facilitation (i.e., the lowest point of the curve) in near periphery. The non-linear fit was conducted using SciPy for Python^48^,^49^. The lognormal function used was:


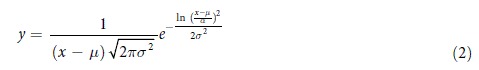


where *μ* represents the value at which the curve reaches its minimum, *σ* corresponds to the shape of the function and *α* is the scale of the function. We estimated the minimum values for the function that best fitted the obtained data. At 1 cpd the minimum values obtained was −0.0046 ± 0.013 s.d. and it was found at 7.52 ± 0.29 s.d. Thus, the peak of facilitation corresponds to a target-to-flankers relative distance of 7.52λ. This is consistent with our previous study in the 1 cpd condition^39^.

**Figure 5:**
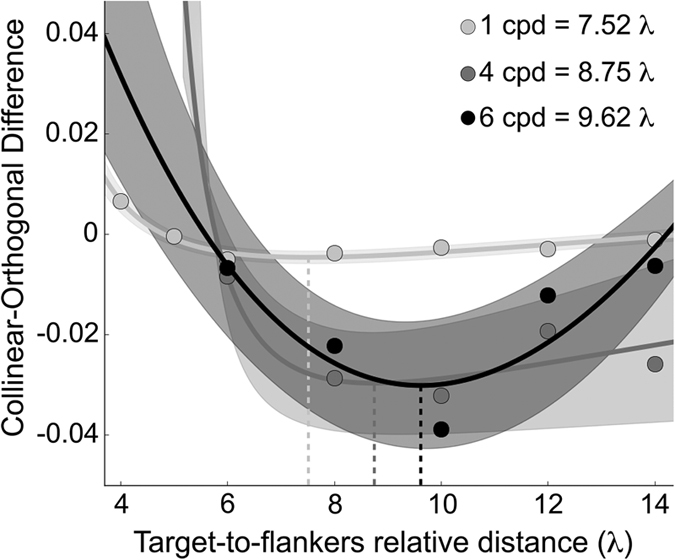
Collinear-orthogonal differences as a function of the target-to-flankers relative distance for 1 (light grey dots), 4 (grey dots) and 6 cpd (black dots) configurations. Lognormal fits are also reported as continuous lines in the corresponding colours whose standard deviations correspond to the surrounding semi-transparent area. Vertical dashed lines correspond to the minimum values of the fitted function for each spatial frequency.

Equation () was also fitted to collinear-orthogonal differences of Experiment 2 in order to estimate the peak of collinear facilitation for spatial frequencies of 4 and 6 cpd. The estimated minimum values were −0.0297 ± 0.009 s.d. at 8.75 ± 0.54 s.d. for 4 cpd and −0.0302 ± 0.011 s.d. at 9.62 ± 0.77 s.d. for 6 cpd. Thus, the peak of facilitation for 4 and 6 cpd was located at a target-to-flankers relative distance of 8.75λ and 9.62λ, respectively. In these two cases, in order to establish more precisely the location of the peak of collinear facilitation, we excluded from the fitting procedure the mean collinear-orthogonal difference in correspondence of 16λ. This is because at 16λ there seems to be some residual collinear facilitation, but contrast thresholds (and consequently collinear-orthogonal differences) obtained at this target-to-flankers relative distance are quite noisy and the collinear facilitation estimated is not statistically significant. To establish the goodness of fit of our data on the obtained lognormal curves, we performed a post-hoc goodness of fit. Our values were quite close to those expected by those predicted by the function (1 cpd: χ^2^  = −0.002, *p* > 0.05; 4 cpd: χ^2^  = −0.0026, *p* > 0.05; 6 cpd: χ^2^ = − 0.012, *p* > 0.05). This corroborates our idea that the lognormal distribution explain quite well the behaviour of the results at different spatial frequencies. Figure 5 shows collinear-orthogonal differences for each spatial frequency and relative fits.

## Discussion

In this study we tested the extent of collinear facilitation for configurations presented at 4° of eccentricity (i.e., near periphery). The rationale was that since previous studies showed that collinear facilitation in the near periphery of the visual field emerges at target-to-flankers relative distances 2–3 times larger than in the fovea^36^,^38^,^39^, the spatial extent of facilitation in the near periphery might be different with respect to the fovea.

In Experiment 1 we used low spatial frequency stimuli (1 cpd) and the results showed collinear facilitation at a target-to-flankers relative distance of 6λ^36^,^39^, decaying at 10λ. Overall, this range is shorter than the typical range of collinear facilitation found in fovea, extending from 1.5–2λ to 12λ^3^,^11^.

In Experiment 2 we used stimuli with higher spatial frequencies (i.e., 4 and 6 cpd). The rationale was that the magnitude of collinear contrast sensitivity in the near periphery seems to differ depending on the spatial frequency used, being weaker for high spatial frequencies^36^,^38^,^39^. Results showed that collinear facilitation emerges at a target-to-flankers relative distance of 8λ for both spatial frequencies, returning to baseline at 12λ (i.e., collinear contrast thresholds not significantly lower than orthogonal contrast thresholds).

Overall, collinear facilitation, defined as the target-to-flankers relative distance at which collinear contrast thresholds are significantly lower than orthogonal contrast thresholds, seems to differ between low spatial frequency (1cpd) and mid-high spatial frequencies (4 and 6cpd) both in terms of spatial range and magnitude of the effect. In particular, based on the curve fitting with the lognormal function, for the lowest spatial frequency facilitation peaked at 7.52λ, while for 4 and 6 cpd, it peaked at 8.75λ and 9.62λ, respectively. Moreover, the facilitatory effect of collinear flankers (expressed as the difference between collinear and orthogonal contrast thresholds) for 1 cpd is one order of magnitude smaller than 4 and 6 cpd, while the suppressory effect at shorter target-to-flankers relative distances is much stronger for 1 cpd than for 4 and 6 cpd.

However, it should be noted that when Polat and Sagi^3^ psychophysically described the phenomenon of lateral interactions and collinear facilitation, they did not perform statistical analysis to support their findings, rather the authors indicated 12λ as the target-to-flankers relative distance at which contrast thresholds return to baseline. Thus, most of these effects have been previously described without a statistical cut-off to define formally the range of foveal facilitation, and this makes difficult to compare our results with previous findings in foveal vision. Accordingly, if we adopt a more descriptive approach, considering the extent of collinear facilitation as the maximum target-to-flankers relative distance at which the difference between collinear and orthogonal contrast thresholds approaches zero, we can observe how collinear thresholds for 4 cpd are on average lower than the orthogonal thresholds for all the target-to-flankers relative distances tested, except for the last one, returning above zero at 16λ. On the other hand, for 6 cpd, collinear thresholds are lower than orthogonal thresholds across the entire range of relative distances tested.

**Figure 6:**
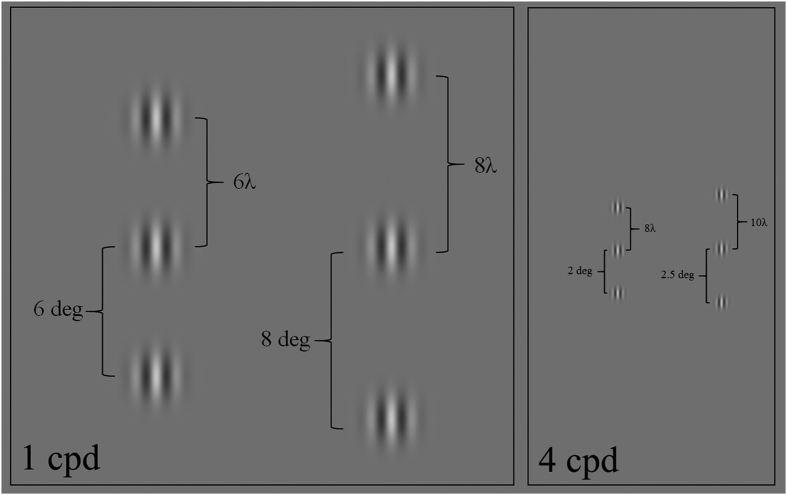
Spatial range of near peripheral collinear facilitation expressed in angular distance (degrees of visual angle) for 1 and 4 cpd. Only the target-to-flankers relative distances for which we showed a significant difference between collinear and orthogonal conditions are reported. Collinear facilitation is scaled for the wavelength of the stimuli used (emerging at 6λ and 8λ for the spatial frequencies shown) independently from the angular distance.

Within this framework, the estimation of 3 deg of spatial extent for collinear facilitation^11^ is too short to explain the effect we found at 8λ with 1cpd stimuli, for which the angular distance between the target and flankers is 8 deg. Similarly, the foveal limit of 12λ is overcome for the highest spatial frequencies tested (i.e., 6 cpd). Interestingly, the peak of collinear facilitation seems to shift rightwards, i.e., towards larger target-to-flankers relative distances, but shorter angular distances. This is because σ = λ, so higher spatial frequencies are characterized by shorter target-to-flankers distances in terms of angular distance. This phenomenon has never been reported in previous studies with foveal or peripheral stimulus presentation. A shorter range of collinear facilitation is expected for lower spatial frequencies because of the neural integration time; that is, neural signals from flankers located at more than 10 deg of visual angle (i.e., 10λ with 1 cpd stimuli) would fail to reach the neuron responding to the target within the contrast integration time window. Polat^6^ showed that the magnitude of collinear facilitation in fovea increases with increasing spatial frequency. The author argued that facilitation for low spatial frequencies is reduced because of the slow propagation speed of the flankers input, failing in combining with the target’s input within the temporal integration window, estimated to be around 200 ms^10^,^50^. Moreover, since collinear facilitation seems involved in contour integration^33^,^51^, the preference for high spatial frequencies can be explained in terms of ecological values, since intermediate and high spatial frequencies are more involved in this process and more trained by everyday life^6^,^52^. However, Maniglia *et al.*^39^ reported how collinear facilitation with stimuli presented in the near periphery of the visual field (4° of eccentricity) seems to show an inverse pattern, with a preference for lower spatial frequencies, consistent with the known spatial frequency tuning of this portion of the visual field^53^. Therefore, it seems also that the temporal integration window and/or the propagation speed are different between the fovea and the near periphery of the visual field. Polat^6^ reported how estimation of cortico-cortical propagation, provided by psychophysics studies^12^,^54^ is about 3 deg/s. However, while these estimations both in terms of temporal integration window and propagation speed may be plausible and consistently verified in foveal vision, they are not suitable to explain the effect observed in the present study, in which we reported statistically significant facilitation at 1 cpd for 8λ and up to 10λ with 4 cpd (Fig. 6).

The angular distance between target and flankers in the classical lateral interaction paradigm is related to the spatial frequency of the Gabor stimuli used (since σ = λ), so in the case of spatial frequency of 1 cpd, a target-to-flankers relative distance of 8λ is four times larger, in terms of visual angle, than a target-to-flankers relative distance of 8λ with 4 cpd stimuli (Fig. 6).

If the integration time is 200 ms and the propagation speed from flankers’ location is 3 deg/s, then for 1 cpd and target-to-flankers relative distance of 8λ, the input from the flankers would arrive at the target location after 2.66 seconds (being the angular distance between flankers and target 8 deg), while for 4 cpd and target-to-flankers relative distance of 10λ (target-to-flankers angular distance of 2.5 deg), it would be 830 ms, that is beyond the integration time window^10^.

However, more systematic studies taking into account the cortical magnification factor, the propagation speed for near peripheral stimulation and the integration time for near peripheral contrast response are needed in order to shed light on this phenomenon.

As reported in the introduction section, one of the proposed anatomical substrate for collinear facilitation are the long-range horizontal connections between units in the primary visual cortex sharing the same orientation selectivity^7^,^21^,^55^,^56^,^57^. Several studies have proven that neurons can synchronize their firing rate with a millisecond precision^58^. This effect was reported for spatially separated units and involves stimuli similar to those used to investigate collinear facilitation. Consequently, it is possible that practice can improve collinear facilitation in the near periphery and uncover a larger range of facilitation also for low spatial frequencies, since previous studies with foveal presentation already showed that collinear facilitation can be strengthened through practice^4^,^5^,^59^,^60^. Interestingly, Polat^6^ also proposed that the propagation time may be slower in the periphery. However, this is somewhat inconsistent with the data presented here, showing that facilitation may arise for target-to-flankers angular distances of 8 deg.

An alternative explanation is that propagation speed is the same for horizontal long-range connection between units coding central and near peripheral vision. In this case, the size of PFs is larger, so that each unit analyses a bigger portion of the visual field and anatomically close neurons in the visual cortex are responsible of a wider portion of the visual field. Therefore, anatomically close neurons can be activated by stimuli located in more distant spatial locations. This is consistent with the definition of the cortical magnification factor^43^. Overall, our data show that when presenting near peripheral stimuli, propagation time is fast enough to lead to the integration of signals coming from the flankers for distances of 8λ with 1 cpd stimuli (i.e., for an angular distance between flankers and target of 8 deg).

However, a strong alternative explanation for the effects we reported would take into account feedback mechanisms from higher-level visual areas. One of the main reasons is the angular distances at which contextual modulations are still present (8 deg). Such large interactions cannot be easily explained in terms of horizontal connections in early visual cortex alone, suggesting the involvement of extrastriate areas, whose units present receptive fields up to 6 times bigger than V1^27^. Moreover, as reported in the introduction section, the retinal extent of contextual modulation in V1 is wider than the area encompassed by the average axonal length alone^28^.

Future psychophysical studies might reveal that this range of collinear facilitation can be extended by practice, promoting synaptic synchronization for larger target-to-flankers relative distances, as already showed for foveal presentation^4^.

A further question that should be addressed is whether the minimum target-to-flankers relative distance necessary to elicit collinear facilitation increases with increasing eccentricity. To our knowledge, collinear facilitation in the periphery of the visual field has been tested mainly at 4° of eccentricity^36^,^38^,^41^,^42^,^61^ and overall not beyond 6° 42. Assuming that the size of PFs increases as a function of eccentricity, we might expect that facilitation emerges at larger target-to-flankers relative distances for more eccentric spatial locations. However, in the case of large angular distances, the propagation of the signal from the flankers to the target might not be fast enough to reach the target location within the integration time window, thus interfering with collinear facilitation. Further studies might address this issue by training subjects on larger target-to-flankers relative distances.

## Additional Information

**How to cite this article**: Maniglia, M. *et al.* The spatial range of peripheral collinear facilitation. *Sci. Rep.* **5**, 15530; doi: 10.1038/srep15530 (2015).

## Figures and Tables

**Table 1:** Summary of the differences between foveal and near peripheral collinear interactions.

Property	Fovea	Near Periphery (max 4° eccentricity)
Collinear suppression	Target-to-flankers relative distance <2λ^3^	Target-to-flankers relative distance up to 4–6λ^38^,^41^,^42^
Collinear facilitation	Target-to-flankers relative distance >2λ^3^	Target-to-flankers relative distance up to 6–8λ^36^,^38^,^39^
Range of lateral interaction	Up to 12λ^3^	Never tested
Electrophysiological evidence	VEPs amplitude maximal for 3λ relative distance, the effect vanishes at 12λ^11^	Never tested
Effect of Perceptual Learning	Perceptual learning reduces short distance suppression and increases long distance facilitation^4^	Perceptual learning mainly reduces short distance suppression^38^
Magnitude of collinear facilitation	Collinear facilitation increases as the spatial frequency increases^6^	Collinear facilitation decreases as the spatial frequency increases^39^
Spatial frequency selectivity	Narrower range of spatial frequencies selectivity for facilitation than suppression^3^	Never tested

**Table 2:** Summary of the statistically significant differences between collinear and orthogonal configurations at 1 cpd.

Target-to-flankers relative distance	*t*_*(9)*_	*Adjusted-p*	Cohen’s d
4λ	3.0	0.035	0.94
6λ	3.79	0.028	1.19
8λ	3.2	0.035	1.01

Post-hoc t-tests were performed using a false discovery rate of 0.05. *d* refers to the Cohen’s d effect size calculated as *d = t/*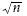, where *t* is the t-value and *n* is the sample size. Cohen’s d of 0.2 represents a small, 0.5 medium and >0.8 large effect size.

**Table 3:** 

Simple Contrasts	*F*_(1,9)_	*p*	*Partial-η*^2^
5λ vs. 4λ	7.88	0.02	0.47
6λ vs. 4λ	17.8	0.002	0.66
8λ vs. 4λ	13.1	0.006	0.59
10λ vs. 4λ	11.45	0.008	0.56
12λ vs. 4λ	17.56	0.002	0.66
14λ vs. 4λ	9.73	0.012	0.52

Summary of the simple contrasts performed between collinear-orthogonal differences calculated at target-to-flankers relative distances of 5λ, 6λ, 8λ, 10λ, 12λ, 14λ with respect to the collinear-orthogonal difference calculated at 4λ.

**Table 4:** Summary of the statistically significant one-sample t-tests between collinear-orthogonal differences and zero.

Target-to-flankers relative distance	*t(9)*	*Adjusted-p*	Cohen’s *d*
4λ	3.01	0.035	0.95
6λ	3.79	0.028	1.19
8λ	3.2	0.035	1.01

One-sample t-tests were performed using a false discovery rate of 0.05.

**Table 5:** Summary of the statistically significant paired t-tests between contrast thresholds estimated for the collinear and orthogonal configurations.

Spatial Frequency	Target-to-flankers relative distance	*t*_*(9)*_	*p*	Cohen’s *d*
4 cpd	8λ	−2.74	0.023	0.86
4 cpd	10λ	−2.37	0.042	0.79
6 cpd	8λ	−2.26	0.05	0.71
6 cpd	10λ	−2.27	0.049	0.70

**Table 6:** Summary of the statistically significant differences (paired t-tests) for collinear-orthogonal differences respect to zero for Experiment 2.

Spatial Frequency	Target-to-flankers relative distance	*t*_*(9)*_	*p*	Cohen’s *d*
4 cpd	8λ	−2.74	0.023	0.87
4 cpd	10λ	−2.37	0.042	0.75
6 cpd	8λ	−2.26	0.05	0.71
6 cpd	10λ	−2.27	0.049	0.72
